# Treatment of enterohemorrhagic *Escherichia coli *(EHEC) infection and hemolytic uremic syndrome (HUS)

**DOI:** 10.1186/1741-7015-10-12

**Published:** 2012-02-02

**Authors:** Paul N Goldwater, Karl A Bettelheim

**Affiliations:** 1 Microbiology and Infectious Diseases, SA Pathology at the Women's and Children's Hospital, and Discipline of Paediatrics, University of Adelaide, 72 King William Road, North Adelaide, South Australia, Australia; 2 K.A.B.: Flat 5, Rosedale Lodge, 220 Chase Side, Southgate, London, N14 4PM, UK

**Keywords:** enterohemorrhagic *Escherichia coli *(EHEC), verotoxigenic (VTEC) hemolytic uremic syndrome (HUS), treatment, pathogenicity, EAHEC

## Abstract

Verotoxigenic *Escherichia coli *(VTEC) are a specialized group of *E. coli *that can cause severe colonic disease and renal failure. Their pathogenicity derives from virulence factors that enable the bacteria to colonize the colon and deliver extremely powerful toxins known as verotoxins (VT) or Shiga toxins (Stx) to the systemic circulation. The recent devastating *E. coli *O104:H4 epidemic in Europe has shown how helpless medical professionals are in terms of offering effective therapies. By examining the sources and distribution of these bacteria, and how they cause disease, we will be in a better position to prevent and treat the inevitable future cases of sporadic disease and victims of common source outbreaks. Due to the complexity of pathogenesis, it is likely a multitargeted approach is warranted. Developments in terms of these treatments are discussed.

See related article: http://www.biomedcentral.com/1741-7015/10/11

## Introduction

The association of verotoxigenic *Escherichia coli *(VTEC) with human disease goes back over 30 years [1-3]. The occurrence of outbreaks due to VTEC in the USA in 1982 [4] focused the world's attention onto these pathogens. Since the discovery of verocytotoxin [1,3], and the paper by Karmali *et al. *[5] of cases of post-diarrheal hemolytic uremic syndrome (D+HUS) caused by VTEC, otherwise known as Shiga-toxigenic *Escherichia coli *(STEC), a large body of knowledge has accumulated, yet despite this information, successful treatment of these infections has remained elusive.

### Sources and pathogenesis of VTEC infection

#### Sources and spread of VTEC

Gut colonization of farm animals, especially ruminants such as cattle, sheep and goats is the likely origin of VTEC/STEC. From these sources derive a variety of vehicles of transmission to humans, including many different foods of animal or plant origin, and water used for swimming and drinking and for growing edible plants. Human fecal contamination of food and seeds could also play a role, especially in developing countries [6].

The potential for VTEC spread is further compounded by globalization of food, which presents a great opportunity for VTEC to spread quickly to large sections of the population. Global food distribution carries an inherent risk and presents great difficulties in controlling food-borne pathogens and in identifying sources of outbreaks, as was recently witnessed in Europe. This is further discussed in the commentary by Werber *et al. *[7].

#### VTEC strains

Various strains of VTEC exist, and, as discussed in the linked commentary, O157 clones, although less prevalent than non-O157 strains, tend to be more virulent. Thus, although non-O157 VTEC strains had originally been reported and continued to be reported, albeit only by dedicated microbiologists, most researchers in the field largely ignored them. No attention appears to have been given to the generally observed fact that there is a widespread diversity of *E. coli *serotypes in the human intestine at any one time [8] and this has also been found in animals, especially cattle [9]. Most ruminant feces contain a variety of VTEC serotypes, but some, such as O157 and also O111, though rarely present and then in only small numbers, are particularly virulent. Importantly, an increasing number of other serotypes can be involved and one study of an outbreak has shown that the more VTEC serotypes with which a patient is infected, the worse the clinical condition [10] (though the main VTEC serotype was O111). While isolations of VTEC O111 from cattle are rare, non-VTEC strains, which are otherwise indistinguishable from the VTEC strains, appear abundant, especially in the feces of sick cattle and patients [11].

Detailed studies [12] have shown that the Shiga toxins can be subdivided into a series of subtypes and that these are also host specific. Thus there is a 'double host specificity' among VTEC strains. Some clones are specific to cattle, while others are specific to sheep. The toxin subtypes these strains carry are specific to the VTEC types found in these mammalian hosts. Therefore, by not looking for the presence of all VTEC serotypes during an outbreak, a great deal of epidemiological information is lost and indication of the source animal is not identified.

#### Pathogenesis of post-diarrheal hemolytic uremic syndrome

VTEC/STEC/enterohemorrhagic *E. coli *(EHEC) belong to clones of zoonotic *E. coli *of different O serogroups. These serogroups have evolved and acquired specific virulence factors that enable the bacteria to colonize and infect the human colon, usually without invasion of the blood stream [13]. Once they have been ingested, STEC/VTEC/EHEC cause bloody diarrhea (BD), severe colitis and HUS. These bacteria are known as EHEC when infection is associated with severe colonic and/or renal disease. The production of Vero/Shiga toxins have been considered the basis for their pathogenicity, however, other toxins such as subtilase cytotoxin (SubAB) [14] and cytolethal distending toxin [15] and secreted protease of C1 esterase inhibitor from EHEC (StcE) probably play a role [16].

The recent outbreak of foodborne *E. coli *O104:H4 in Europe has once again drawn attention to STEC or EHEC infections together with their devastating complications of renal failure (through HUS), and stroke from intravascular coagulopathy and vasculopathy or thrombotic microangiopathy. The unusual virulence and lethality of the O104:H4 strain is the result of genetic admixture of virulence factors, including enteroaggregative properties and multiple antibiotic resistance, and is a lesson in microbial evolution and the genomic plasticity of *E. coli *[17]. The O104:H4 strain is now known as an enteroaggregative and enterohemorrhagic *E. coli *(EAHEC).

We have recently observed the combined properties of enteroaggregative ability (providing strong attachment via fimbriae and colonization of the colonic epithelium) with Shiga toxin (Stx) production in the novel and highly lethal European *E. coli *O104:H4 strain. It has since been shown that this strain belonged to an enteroaggregative *E. coli *lineage that had acquired genes for Shiga toxin 2 and antibiotic resistance [18].

The pathogenesis of HUS disease remains incompletely understood; remarkably, during HUS serum Stx is undetectable. It seems polymorphonuclear leukocytes (PMN) are key players in delivering Stx to critical sites such as the kidneys. The extent of renal damage in children with STEC-associated HUS may relate to the concentration of Stx present on circulating PMN [19]. Paradoxically, patients with high amounts of Stx on PMN showed preserved or slightly impaired renal function (incomplete form of HUS), whereas cases with low amounts of PMN-Stx usually present with acute renal failure. Moreover, high amounts of PMN-Stx induce a reduced release of cytokines by the renal endothelium, with congruent lower degree of inflammation, while low toxin PMN amounts trigger a cytokine cascade, provoking inflammation with consequent tissue damage. The microvasculature plays an important role in pathogenesis: D+HUS is associated with platelet thrombi in the microvasculature of almost all vascular beds [20]. Plasma from HUS patients induces apoptosis of cultured microvascular endothelial cells from most organs [21]. Two key events are involved in the pathogenesis of D+HUS: altered Von Willebrand factor (VWF) activity (for example, as seen with 'a disintegrin and metalloproteinase with thrombospondin motif-13' (ADAMTS13) deficiency) and site-specific activation and/or apoptosis of microvascular endothelial cells. A deficiency in ADAMTS13, which mediates proteolytic processing of newly released proadhesive ultralarge VWF multimers from endothelial cells, is also thought to play a role in D+HUS coagulopathy [22]. Targeting the interruption of these processes gives hope for potential novel treatment modalities.

Bacterial gut pathogens target the follicle-associated epithelium overlying Peyer's patches. The microorganisms breech the intestinal barrier via M cells and are captured by mucosal macrophages [23]. STEC/EHEC are able to interact *in vivo *with Peyer's patches and translocate through the mucosa. After being taken up by macrophages and M cells the bacteria produce Stx and induce apoptosis of these host cells and Stx release. These microbe/host cell interactions could represent new therapeutic targets [23].

#### Current treatment strategies: a multitargeted approach

HUS comprises acute renal failure and its consequential perturbation of fluid and electrolyte balance, hemolysis, disruption of the clotting cascade with thrombocytopenia, with the risk of stroke. This syndrome, together with the further effects of toxin, and complement complex formation, must be managed and addressed urgently using a multitargeted approach. This involves the institution of general supportive measures, antiplatelet and thrombolytic agents and thrombin inhibitors, selective use of antimicrobials, probiotics, toxin neutralizers (synthetic and natural binders, antibodies, and so on); and antibodies against key pathogenetic pathway elements to interrupt pathological processes (for example, inhibition of terminal complement complex formation). Targeting PMNs carrying Stx could be a productive strategy for future research, as could possible gene therapy. The management of D+HUS is complex by virtue of the nature of the condition and the variety of pathways affected. Table 1 summarizes the approach to management and lists trialed and experimental treatments.

**Table 1:** Approach to management: summarizing trialed and experimental treatments.

Problem	Treatment	Detail and comments	refs
Fluid and electrolyte imbalance	Intravenous fluids	Fluid balance and attention to the volume and sodium content of intravenous fluids administered early in the disease have been shown to reduce the risk of developing oligoanuric HUS after *Escherichia coli *O157:H7 infections	[25]
		Intravenous fluids within first 4 days of onset of diarrhea (isotonic preferable). The overall oligoanuric rate of the 50 participants was 68%, but was 84% among those not given intravenous fluids in the first 4 days of illness. The relative risk of oligoanuria when fluids were not given in this interval was 1.6 (95% CI, 1.1 to 2.4; *P *= 0.02). Children with oligoanuric HUS were given less total intravenous fluid (r = -0.32; *P *= 0.02) and sodium (r = -0.27; *P *= 0.05) in the first 4 days of illness than those without oligoanuria.	[24]

Acute renal failure	Acute renal replacement therapy	Peritoneal dialysis (safe with thrombocytopenia) Hemodialysis Plasma infusion and plasma exchange	[26] [27] [28]
	Apheresis	Uncertain benefit	[29]
	Antihypertensives	Where indicated	[13,30]

Hematological: hemolytic anemia		Transfusion (packed red cells)	[13]

Hematological: thrombocytopenia		Platelet transfusion (usually avoided)	[13,30]

Preventing further effects of toxin	Antibiotics	Generally to be avoided because of VT/Stx/endotoxin release from dying/dead bacteria. β-lactams to be avoided. Subinhibitory levels may increase toxin production/release	[13] [39] [32]
		The quinolone ciprofloxacin but not fosfomycin causes Shiga toxin-encoding bacteriophage induction and enhanced Stx production from *E. coli *O157:H7 *in vitro *and *in vivo *in a mouse model.	[33,34]
		Fosfomycin showed evidence of better outcomes in a mouse-model of STEC infection and was recommended for human studies. Similar results were observed in a gnotobiotic piglet model. Pooled prospective data showed no benefit of antibiotics There is only a single study purportedly connecting fosfomycin with a reduced risk of HUS	[35]
		Fosfomycin benefit in humans remains in doubt. The validity of the study has been questioned on the basis that the meta-analysis mischaracterized fosfomycin as being superior to no antibiotics.	[36]
	Lumenal toxin neutralisers: Synthetic ligand mimics	Synsorb K; trial showed no benefit	[37]
	Modified bacteria decorated with Gb3 or Gb4 Super Twig (Gb3 polymer)	Not yet trialed Clinical trials awaited	[40]
	Antibodies: Monoclonal against A subunit	Protective in lethally-challenged animals	[41]
	Oral bovine colostrum	No effect on complications; decreases stool frequency but not STEC carriage	[42]
	LPS antibodies	Reduces *in vitro *adherence. No human data. Experimental only.	[43]
	Receptor blockers and toxin intracellular transport inhibitors	Ac-PPP-tet blocks intracellular transport of Stx2 from Golgi to endoplasmic reticulum (essential for Stx2 toxicity) Watanabe-Takahashi *et al. *reviewed other neutralizers that do not act on receptor binding but disrupt intracellular transport of the toxin, effectively neutralizing the toxin.	[45]
	Systemic (intravenous) toxin binders		[46] [47]
		Cell-permeable peptide binds to Stx2 and prevents acute kidney injury. Increases survival in juvenile baboon model. TVP (5 mg/kg) delivered intravenously and simultaneously with toxin or at 6 or 24 h after toxin with daily 1 mg/kg supplements up to day 4 prevented acute kidney injury and	[47]
		delayed and reduced blood urea and creatinine levels and increased survival. Delayed administration of the peptide significantly reduced thrombocytopenia, but had no effect on anemia. This cell-permeable agent shows promise in counteracting Stx2 lethality in a baboon model; outcomes of human trials are awaited.	[48]
	Blockers of endosome-to-Golgi trafficking of Stx	Recently it was shown that the metal manganese (Mn^2+^) blocks endosome-to-Golgi trafficking of STx and causes its degradation in lysosomes. Mn2+ targets the cycling Golgi protein GPP130. Direct trafficking of STx from early endosomes to the Golgi, (bypassing late endosomes and lysosomes), is a crucial step that allows STx to avoid degradation. Mn^2+^, as a small-molecule inhibitor targeting this step therefore offers a cheap therapeutic modality given that mice injected with nontoxic doses of Mn2+ were completely resistant to a lethal STx challenge.	[49] [49]

Blockers of bacterial and host cell interaction	Probiotics	Harmless recombinant bacteria expressing surface molecules that mimic host cell receptors, deceiving pathogen into attaching to probiotic cell rather than the host cell receptor. Unlikely to benefit symptomatic patients but could be beneficial as prophylactic for family and close contact/exposed persons. Supernatant of cultures of *Bifidobacterium longum *HY8001 is designed to inhibit the effect of VT/Stx through interference of B subunit of VTs in binding to Gb3.	[50]

Terminal complement complex formation	Eculizumab (intravenous)	This monoclonal antibody blocks activation of complement and Factor H binding via alternative pathway.	[51,52]
		Promising results in small clinical pilot study. The antibody was given intravenously at 7 day intervals, twice in two patients and four times in a third patient.	[53]

Immunoprophylaxis	Vaccines	Promising results in animal studies using:	
		(1) virulence proteins (Stx1/2, intimin, EspA; peptides;	[54] [55]
		fusion proteins of A and B Stx subunits);	[56]
		(2) avirulent ghost cells of EHEC O157:H7;	[57]
		(3) live attenuated bacteria expressing recombinant proteins. Gu *et al. *used a live attenuated EIS-producing *Salmonella *vaccine in mice model. Vaccination induced significant increases of EspA, intimin and Stx2 specific IgG in serum and secretory IgA in feces as well as antigen-specific T cell proliferation;	[58]
		(4) recombinant fimbrial proteins have been developed in a quest to protect against the STEC-related entity piglet edema disease. Early results are mixed.	[59]
		Tir, EspB, EspD, NleA, and EspA were highly immunogenic in vaccinated and naturally infected subjects and represent future candidates for a STEC vaccine;	[60]
		(5) DNA vaccines: EHEC Stx2 A2 and B subunits confer immunity in a mouse model;	[61]
		(6) plant-based oral recombinant Stx2 vaccine protects mice.	[62]

EHEC = enterohemorrhagic *Escherichia coli*; EspA/B/D = *E. coli *secreted protein A/B/D; Gb3 = globotriaosylceramide; Gb4 = globotetraosylceramide; HUS = hemolytic uremic syndrome; LPS = lipopolysaccharide; NleA = non-LEE-encoded effector A; STEC = Shiga-toxigenic *Escherichia coli*; Stx = Shiga toxin; Tir = translocated intimin receptor; VT = verotoxin.

#### General supportive measures

Fluid levels and electrolyte balance are extremely important in preventing and managing the development of HUS [24,25] (See Table 1).

Acute renal replacement therapy (ARRT); for example, peritoneal dialysis (PD) or hemodialysis) has been shown to improve outcomes. Children with D+HUS and acute kidney injury given early PD may have improved outcomes without risk of bleeding in patients with low platelet counts. Moreover, the procedure seems safe especially in cases with very low platelet counts with no bleeding episodes recorded [26]. Alternatively, hemodialysis is often necessary. Antihypertensive therapy for hypertension when appropriate is also necessary. There seems to be a beneficial role for plasma infusion [27] and plasma exchange [28], however, benefit from apheresis remains uncertain [29].

#### Managing hematological issues and coagulopathy

Monitoring of hemoglobin, hematocrit and platelet count is essential. Monitoring hemolysis with lactate dehydrogenase (LDH) and haptoglobin is also helpful. Anemia resulting from hemolysis may need correction with transfusions of whole blood or packed red cells. Platelet transfusion is rarely required and usually avoided [13,30].

### Preventing the further effects of toxin

#### Antimicrobials: to use or to avoid?

Due to the potential for undesirable release of verotoxin (VT)/Stx by dying and dead bacterial cells, antibiotics are usually avoided [31]. In addition, the risk of endotoxin release could add to the patient's already potentially lethal burden. *In vitro *subinhibitory concentrations of antibiotics may increase production and release of VT/Stx [32] via bacteriophage induction [33]. A mouse [34] and piglet study [35] suggested human trials of fosfomycin were warranted. However, pooled prospective data showed no benefit of antibiotics [36]. Only one fosfomycin trial has been reported [37]. However, fosfomycin data has been questioned [38] (See Table 1). While many doctors in Japan still use antibiotics including fosfomycin in patients with definite or possible enteric STEC infections the prevailing consensus elsewhere indicates antibiotics should be avoided [13]. More recent evidence supports this especially in relation to β lactam and other bactericidal antibiotics [39].

#### Lumenal toxin neutralizers (synthetic and natural binders, antibodies, and so on)

Strategies using ligand mimics of the receptor for Stx, globotriaosylceramide (Gb3), binding to Stx in the gastrointestinal tract with the intention of preventing the spread of toxin to extraintestinal sites have been proposed. However, in clinical practice the damage has already been done before these ligands could be of benefit. Only one clinical trial has been conducted (alas unsuccessfully) with one agent, Synsorb PK, which bore out this fact [40]. Other agents are listed in Table 1[41,42].

Intralumenal neutralizers might be effective in reducing systemic uptake of toxin but because the toxin is purportedly not found in serum, studies designed to examine the effect of neutralizers on the toxic effects of polymorphonuclear leukocyte-associated toxin would be a first step.

#### Antibodies

Neutralizing Shiga toxin-specific antibodies are potentially useful as therapeutic agents. The toxins are AB toxins with active and binding elements and are obvious targets for antibody neutralization. Monoclonal antibodies targeting the A subunit epitopes of Stx1 have been shown to be highly protective, when administered to lethally treated animals [43]. Orally administered immunoglobulin has been used therapeutically for a number of gastrointestinal infections (for example, rotavirus; Gastrogard-R) [44]. Patients with diarrhea caused by diarrheagenic *E. coli*, specifically STEC and *E. coli*-expressing intimin and HEC-hemolysin were treated by administration of pooled bovine colostrum, rich in antibodies to Shiga toxin and enterohemorrhagic *E. coli*-hemolysin, in a placebo-controlled, double-blind study. Symptom resolution and fecal excretion of infecting strains were assessed. No effect of colostrum therapy on the carriage of the pathogens or on complications of the infection could be demonstrated, however, stool frequency was reduced [45]. Antibody to *E. coli *lipopolysaccharide (LPS) also has the potential of therapeutic use through its blocking effect on adherence of STEC to the human intestinal epithelial (Henle 407) cell line [46]. Likewise, human trials would be needed to show clinical effectiveness.

#### Other toxin binders/neutralizers

Most of these agents bind to toxin directly and inhibit the binding to its receptor present on the target cells [47]. Such novel Stx neutralizers offer a new therapeutic modality against STEC/EHEC infections [47] and are detailed in Table 1.

#### Systemically-applied (intravenous) toxin binders

A cell-permeable peptide (TVP) that binds to Stx2 was shown to reduce disease severity and rescue juvenile baboons from a lethal Stx2 dose (50 ng/kg) [48].

#### Blockers of endosome-to-Golgi trafficking of Stx

Recently it was shown that the metal manganese (Mn^2+^) blocks endosome-to-Golgi trafficking of STx. [49] This offers a possible cheap therapeutic approach. (Table 1).

#### Blockers of bacterial and host cell interaction: probiotics

Gut pathogens display surface molecules enabling the organism to bind to host cell receptors. Similarly, bacterial toxins require host cell receptors for binding and cell entry. To block microbe and host cell interaction 'designer' probiotics have been developed. The harmless recombinant bacteria express molecules that mimic host cell receptors (for example, Gb3) on their surface, thereby deceiving the pathogen into attaching to the probiotic rather than the host cell receptor. Probiotic bacteria must survive the tube journey encountering digestive enzymes and other adverse conditions. Trial data is awaited.

A different approach has used the supernatant of cultures of *Bifidobacterium longum *HY8001, designed to inhibit the effect of VT/Stx through interference of B subunit of VTs in binding to Gb3 [50].

#### Inhibition of terminal complement complex formation

Based on evidence that Shiga toxin activates complement and binds factor H and evidence for an active role of complement via the alternative pathway in diarrhea-associated hemolytic uremic syndrome [51,52], a few anecdotal reports of successful treatment of severe Stx-associated HUS with the monoclonal antibody eculizumab have been published [53]. Neurologically, the three patients improved dramatically within 24 h after the first eculizumab infusion. Clinical improvement was associated with rapid normalization of markers of disease activity. These initial results are extremely promising and outcomes from large-scale randomized placebo-controlled trials are optimistically awaited.

#### Vaccines

Several vaccine strategies have been used with variable success in a number of animal models. The strategies have involved the use of recombinant virulence proteins such as Stx, intimin and *E. coli *secreted protein A (EspA) [54] or peptides [55] or fusion proteins of A and B subunits of Stx2 and Stx1 such as Stx2Am-Stx1B [56] or avirulent ghost cells of EHEC O157:H7 [57]. The application of live attenuated bacteria such as *Salmonella *as a carrier for vaccine proteins against mucosal pathogens including EHEC have obvious advantages [58]. Other approaches are listed in Table 1[59-62].

Antibodies produced in humans with HUS and in rabbits immunized with type III secreted proteins (T3SPs) from four STEC serotypes, and experimentally infected cattle revealed proteins common to several HUS serotypes [60] (Table 1). These were highly immunogenic in vaccinated and naturally infected subjects and represent future candidates for a STEC vaccine (Table 1).

As well as protein-based vaccines, DNA vaccines are a recent development in EHEC prevention, providing encouraging results in a mouse model [61] (Table 1).

The mode of administration (intramuscular, intranasal, oral, intragastric, and so on) for a number of these vaccines not only affects immunogenicity but also protective effect under challenge. Vaccination with a plant-based oral vaccine protected mice against lethal systemic intoxication with Stx2 [62]. This is seen as encouraging. Clearly there is some time to go before human trials are reported but the numerous and frequent outbreaks of EHEC disease constantly remind us of the urgent need to protect the population against these emerging and often devastating zoonoses.

#### Future directions and conclusions

There remain significant barriers to successful treatment of HUS given the complexity of the pathogenesis of HUS, which involves perturbation of key homeostatic pathways involving complex biochemical and physiological systems. It is unlikely that targeting a single pathway with a treatment modality will be sufficiently successful; a multitargeted approach would seem necessary. However, given the apparent success of eculizumab, albeit with tiny case numbers, it could offer a promising strategy for treatment. Treatment is designed to prevent the most serious complications of STEC infection (that is, renal failure and central nervous complications, for example, stroke, and shock), which remain far too common. It is clear that a better understanding of the pathogenesis of HUS will lead to additional and possibly better targets for treatment. The discovery that Mn^2+ ^can block endosome-to-Golgi trafficking will no doubt lead to randomised controlled trials in humans. These will be awaited with keen interest. In terms of prevention, we should question the globalization of food distribution with its inherent dangers and its wasteful use of energy resources resulting in a giant carbon footprint.

## Abbreviations

EHEC: enterohemorrhagic *Escherichia coli*, VTEC: verotoxigenic *Escherichia coli*; STEC*; *Shiga-toxigenic *Escherichia coli; *HUS: hemolytic uremic syndrome; EAHEC: enteroaggregative and enterohemorrhagic *E. coli; *VT: verotoxin; Stx: Shiga toxin; D+HUS: post-diarrheal hemolytic uremic syndrome; ARRT: Acute renal replacement therapy; PD: peritoneal dialysis;

## Competing interests

The authors declare that they have no competing interests.

## Authors' contributions

Both authors read and approved the final manuscript.

## Authors' information

PNG and KAB have been involved in STEC/EHEC research for over 30 years. PNG is a senior consultant clinical microbiologist and infectious diseases physician and with his collaborators has used his professional knowledge to further develop an understanding of STEC infection in children. KAB, now retired, introduced PNG to the fascinating field of *E. coli *microbiology and both have been close colleagues and research collaborators over many productive years.

## Pre-publication history

The pre-publication history for this paper can be accessed here:

http://www.biomedcentral.com/1741-7015/10/12/prepub
